# Induction of programmed cell death in *Trypanosoma cruzi* by *Lippia alba* essential oils and their major and synergistic terpenes (citral, limonene and caryophyllene oxide)

**DOI:** 10.1186/s12906-018-2293-7

**Published:** 2018-07-27

**Authors:** Érika Marcela Moreno, Sandra Milena Leal, Elena E. Stashenko, Liliana Torcoroma García

**Affiliations:** 1 Infectious Disease Research Program, Universidad de Santander, 680006 Bucaramanga, Colombia; 2 Bacteriology and Clinical Laboratory Program, Universidad de Santander, 680006 Bucaramanga, Colombia; 3 National Research Center for Agroindustrialization of Aromatic Medical and Tropical Species (CENIVAM), Universidad Industrial de Santander, 680002 Bucaramanga, Colombia

**Keywords:** *Lippia alba*, Essential oils, Citral, Caryophyllene oxide, Limonene, Synergy, *Trypanosoma cruzi*

## Abstract

**Background:**

Chagas Disease caused by *Trypanosoma cruzi* infection, is one of the most important neglected tropical diseases (NTD), without an effective therapy for the successful parasite eradication or for the blocking of the disease’s progression, in its advanced stages. Due to their low toxicity, wide pharmacologic spectrum, and potential synergies, medicinal plants as *Lippia alba*, offer a promising reserve of bioactive molecules. The principal goal of this work is to characterize the inhibitory properties and cellular effects of the Citral and Carvone *L. alba* chemotype essential oils (EOs) and their main bioactive terpenes (and the synergies among them) on *T. cruzi* forms.

**Methods:**

Twelve *L. alba* EOs, produced under diverse environmental conditions, were extracted by microwave assisted hydrodistillation, and chemically characterized using gas chromatography coupled mass spectrometry. Trypanocidal activity and cytotoxicity were determined for each oil, and their major compounds, on epimastigotes (Epi), trypomastigotes (Tryp), amastigotes (Amas), and Vero cells. Pharmacologic interactions were defined by a matrix of combinations among the most trypanocidal terpenes (limonene, carvone; citral and caryophyllene oxide). The treated cell phenotype was assessed by fluorescent and optic microscopy, flow cytometry, and DNA electrophoresis assays.

**Results:**

The *L. alba* EOs displayed significant differences in their chemical composition and trypanocidal performance (*p* = 0.0001). Citral chemotype oils were more trypanocidal than Carvone EOs, with Inhibitory Concentration 50 (IC_50_) of 14 ± 1.5 μg/mL, 22 ± 1.4 μg/mL and 74 ± 4.4 μg/mL, on Epi, Tryp and Amas, respectively. Limonene exhibited synergistic interaction with citral, caryophyllene oxide and Benznidazole (decreasing by 17 times its IC_50_) and was the most effective and selective treatment. The cellular analysis suggested that these oils or their bioactive terpenes (citral, caryophyllene oxide and limonene) could be inducing *T. cruzi* cell death by an apoptotic-like mechanism.

**Conclusions:**

EOs extracted from *L. alba* Citral chemotype demonstrated significant trypanocidal activity on the three forms of *T. cruzi* studied, and their composition and trypanocidal performance were influenced by production parameters. Citral, caryophyllene oxide, and limonene showed a possible induction of an apoptotic-like phenotype. The best selective anti-*T. cruzi* activity was achieved by limonene, the effects of which were also synergic with citral, caryophyllene oxide and benznidazole.

## Background

Chagas Disease is one of the most important Neglected Tropical Diseases (NTDs) worldwide, and is one of the most relevant public health problems in Latin America. This infection, caused by the hemoflagellated protozoan *Trypanosoma cruzi*, currently affects an estimated 7 million people in the world, with around 99% of all registered cases occurring in Central and South American countries [1]. The global costs of this disease are calculated at approximately USD $7.19 billion per year [2], with regional economic losses of almost US $1.2 billion, annually [3]. In Colombia, the prevalence of this trypanosomiasis is estimated to be within a range of 700,000 – 1,200,000 cases, with more than 8,000,000 persons at risk [4].

In regions where the condition is endemic, disease-control efforts principally centered on preventing or reducing the *T. cruzi* transmission cycle by vector eradication and massive blood donation screening [5]. However, bigger challenges remain; in particular, those associated with the changing epidemiological profile of the infection (diversity of vectors, reservoirs, and modes of transmission), being the most significant problem, the lack of effective therapies to cure the *T. cruzi* infection or to prevent the progression of the disease, principally in advanced stages.

At present, the conventional Chagas Disease treatments are etiologic, and are comprised of only two possible options, Nifurtimox (NFX) (Lampit®, Bayer) and Benznidazole (BNZ) (Rochagan® in Brazil and Radanil® in Argentina, Roche). These two treatments have remained the standard since their introduction into clinical therapy more than 40 years ago [6]. As disadvantages, these treatments are highly toxic (often accompanied by serious side effects like digestive intolerance, severe anorexia and neurological disorders) [7]; involve prolonged treatment times; and demonstrate variable trypanocidal effectiveness in acute stage (with about 80% being associated with natural resistance). They also display limited efficacy in the late phase of the infection (in which the benefits of these therapies have not clearly defined) [8, 9].

In general, these conventional therapies do not take into account the complex cascade of cellular events leading to Chagasic cardiomyopathy, which are not only associated with the parasite’s presence, but also involve exacerbated and persistent immune response (with cellular and neuronal damage) [10, 11]. These latter factors are those which govern the microvasculopathy and cardiac failure associated with the condition [10, 11].

In this regard, the research and development of new alternative therapies for Chagas Disease remain pressing concerns. New pharmacological approaches should be more efficient and selective, seeking complete parasite elimination, but with adequate modulation of the host immune response and limitation of cellular damage [11]. To this end, in the last two decades, intensive research has been focused on the study of the properties of whole extracts or compounds isolated from plants or synthesized based on natural prototypes, which have shown promising results against parasite infections [12, 13].

Essential oils (EOs) extracted from aromatic plants and their main components have been described as broad-spectrum antimicrobial agents [14], with significant anthelmintic and antiprotozoal activity [15, 16]. Some terpenes of these oils such as citral (*Lippia alba* and *Cymbopogon citratus*), caryophyllene oxide (*Aframomum sceptrum, Achillea millefolium,* and *Piper var brachypodon*), and limonene (*L. origanoides* and *L. pedunculosa*) have demonstrated efficient trypanocidal activity on extra and intracellular forms of *T. cruzi* [17–20]. In addition, these terpenes have been found to exhibit other interesting biological properties, such as being anti-inflammatory immunomodulators, selective antioxidants, and cytoprotectors [21–24]. The presence of citral, caryophyllene and limonene has been identified in EOs isolated from two chemotypes (Citral and Carvone) of the aromatic shrub *Lippia alba* (Miller) N.E. Brown (Verbenaceae), that grows in the Colombian province of Santander [21, 25]. *L. alba* represents the seventh species most cited in traditional Brazilian medicine [26]. The “healers” use their leaves as an infusion to treat health problems such as hypertension, digestive, colds and local wound healing [27, 28]. In the state of Boyacá, Colombia, it is frequently used as an analgesic, for digestive (diarrhea, stomach pain) and respiratory problems (flu and cough) [29]*.* Previous screening studies with these oils evidenced selective inhibition and cytotoxicity against trypanosomatid parasites, in vitro [30]. Taking into consideration their numerous functions, *L. alba* EOs and their bioactive terpenes are a promising platform for development of holistic therapies to combat Chagas Disease. This kind of approach could allow for a selective eradication of the parasite, with less toxicity (even with chemoprotection), and for controlling the host immune response, through a possible synergistic interaction of the compounds involved [22, 31, 32].

The principal goal of this work is to characterize the inhibitory properties and cellular effects of the Citral and Carvone *L. alba* chemotype EOs and their main bioactive terpenes on *T. cruzi* epimastigotes, trypomastigotes, and amastigotes cyclic forms. The IC_50_ was determined for each of these compounds, and possible pharmacologic interactions were defined by a matrix of combinations of the trypanocidal compounds (from Carvone chemotype: limonene and carvone; and from Citral chemotype: citral and caryophyllene oxide). The phenotype of the parasites and mammal cells treated with EOs or terpenes was followed by fluorescent and optic microscopy, flow cytometry, and DNA electrophoresis assays.

## Methods

### Plant material

In this study, specimens of the Citral and Carvone chemotypes of *Lippia alba* (Miller) N. E. Brown (Verbenaceae) were planted in the National Research Center for Agroindustrialization of Aromatic Medical and Tropical species (CENIVAM, in Spanish) located in Bucaramanga, Santander, Colombia, at an altitude of 960 m above sea level. The formal identification of the plant specimens used in this study was provided by Prof. Jorge Luis Fernández Alonso and the vouchers were deposited at the Colombian National Herbarium (Universidad Nacional de Colombia) under Herbarium Codes COL480750 and COL512077, for Carvone and Citral chemotypes of *L. alba*, respectively. A range of environmental and production conditions were used in order to produce 76 EOs with possible diversity in their main compounds. In this regard, the vegetal material was grown, collected, and extracted under the following factors. 1) season: defined as dry (January to March, 26.3 °C temperature, with 68.9% relative humidity, and 1.05 mm/day precipitation) and rainy (April to November, mean temperature of 24.5 °C, relative humidity of 81.3%, and 4.13 mm of daily precipitation); 2) *L. alba* chemotypes (53 from Carvone and 23 from Citral); 3) part of plant harvested (root, stem, fresh and mature leaves, and flowers); 4) vegetal material conditions (fresh and dry); and 5) extraction time (from 30 to 90 min).

### Essential oils extraction and characterization

The oil extraction was performed by microwave-assisted hydrodistillation (MWHD), as described elsewhere [25, 29]. Briefly, a domestic microwave oven (Kendo, 2.45 GHz, 800 W) was modified with a side orifice through which an external Dean-Stark trap joined a round flask that contained the plant material (100 g) and water (0.5 L), inside the oven. Three 15 min heating periods at full power were used to perform the hydrodistillation. The Dean-Stark trap permitted to decant the essential oil from the condensate. A gas chromatograph GC 7890 (Agilent Technologies, AT, Palo Alto, CA, U.S.A.) coupled to a mass selective detector MSD 5975C (AT, Palo Alto, CA, U.S.A.), using electron impact ionization (EI, 70 eV) was used for essential oil characterization. This system included a split/splitless injector (1:30 split ratio), and a MS-ChemStation G1701-DA data system, with the WILEY, NIST and QUADLIB 2007 spectral libraries. For their GC-MS assays, individual essential oil samples (50 μL) were mixed with *n*-tetradecane (2 μL, internal standard) and diluted with dichloromethane to a final volume of 1.0 mL. Helium (99.9995%) was used as the carrier gas, with 155 kPa column head pressure and 27 cm s^− 1^ linear velocity (1 mL minute-1, at constant flow), in two columns of different polarities (DB-5MS and DB-WAX from J&W Scientific, USA). The GC oven temperature was programmed from 50 °C (5 min) to 150 °C (2 min) at 5 °C min^− 1^, then to 230 °C (10 min) at 5 °C min^− 1^. When the DB-5MS column was used, a final heating to 275 °C (15 min) at 10 °C min^− 1^ was added. The temperatures of the injection port, ionization chamber and of the transfer line were set at 250, 230 and 285 °C, respectively. For the polar DB-WAX column, the transfer line temperature was set at 230 °C. Mass spectra and reconstructed (total) ion chromatograms were obtained by automatic scanning in the mass range *m/z* 30–400 at 4.5 scan s^− 1^. Compound relative abundances were calculated from the chromatographic area of profiles obtained with an AT 7890 gas chromatograph provided with flame ionization detection (FID, 250 °C). The chromatographic columns, carrier gas and oven temperature programs employed in GC-FID analysis were the same as described previously for the GC-MS system. The EO compounds were identified using mass spectra and linear retention indices relative to C_8_-C_32_ n-alkanes [33]. Several terpenoid standard compounds, such as limonene, carvone, geranial, geraniol, β-caryophyllene, and β-caryophyllene oxide, obtained from Sigma-Aldrich (St. Louis, MO, U.S.A., with purities above 98%) were used. The extracted EOs were preserved at 4 °C and protected from light before GC-MS and cellular analysis. Finally, the 76 EOs were arranged into 12 groups, according to the significant differences in the percentages of their major terpenes and one oil of each group was arbitrarily selected for further biological analysis, comprising six EOs from Citral chemotype (A13, A20, A23, A24, A25, and A28), and six from the Carvone chemotype (B7, B16, B37, 2B8, 2B18, and 2B19).

### Terpenes and drugs

The terpenes S (+) carvone, D (+) limonene, (−) caryophyllene oxide, and citral were purchased from Sigma-Aldrich (St. Louis, MO). The reference medication BNZ (Radanil®, Roche) was donated by Santander’s State Secretary of Health, and purified by Dr. Leonor Yamile Vargas, from the Environmental Chemistry Program at Universidad Santo Tomás de Aquino (Bucaramanga). Concentrations ranging from 1.85 to 50 μg/mL were used for epimastigote (Epi) and amastigote (Amas) assays and 0.39 to 3.12 μg/mL for trypomastigote (Tryp) assays. The oils (at a density of 0.9 g/mL) and terpene stock solutions were prepared in dimethyl-sulfoxide (DMSO, Sigma-Aldrich (St. Louis, MO)), to get a 10% (*v*/v) solution, without exceeding a DMSO final concentration of 0.1%, in any solution. Working solutions (3.7 to 300 μg/mL) were diluted immediately prior to use with Liver Infusion Tryptose (LIT, Becton Dickinson, FL, USA) media and Dulbecco’s Modified Eagle’s Medium (DMEM, Life Technology, CA, USA) for *T. cruzi* cells and Vero lineage, respectively.

### Cell cultures

Vero lineage derived from African Green Monkey Kidney (Vero, ATCC CCL-81) was used for selectivity index determination and for Tryp and Amas production. These cells were grown on DMEM (Life Technology, CA, USA) media, pH: 7.4; supplemented with 10% of inactivated Fetal Bovine Serum (FBSi), 1000 U/mL of penicillin, and 100 μg/mL of streptomycin; and incubated at 37 °C with 90% humidity and a 5% CO_2_ atmosphere. Epi of *T. cruzi* I (TcI) SYLVIO-X10 strain, were donated by Dr. Marcos López-Casillas, from Fundación Cardiovascular de Colombia and grown in LIT medium (Merck) supplemented with 10% FBSi, and incubated at 28 °C. The Trypomastigotes Derived from Cells (TDC) were obtained by infection of a confluent monolayer of Vero cells with 12 day-old stationary growth phase Epi and incubated under the same conditions described above for Vero cells.

### Cytotoxic activity on Vero cells

Vero cells (3 × 10^5^ cel/mL) were incubated at 37 °C in a 5% CO_2_ atmosphere and at 95% humidity for 24 h to ensure the formation of a confluent monolayer. After this time, the cells were treated with the EOs or their terpenes in four different concentrations (11.1, 33.3, 100, and 300 μg/mL). Thereafter, the lineages were incubated for 70 h at 37 °C in a 5% CO_2_ atmosphere and re-incubated 2 more hours with WST-1 (Roche, Mannheim, Germany), after which Optical Density (OD) measurements were analyzed by spectrophotometry. The cytotoxicity percentage was calculated using [(OD_450nm_ Control – OD_450nm_ treatment) / OD_450nm_ treatment)] × 100. The results were expressed as Cytotoxic Concentration 50 (CC_50_).

### Anti-parasitic activity on *T. cruzi*

*T. cruzi* Epi (5 × 10^5^ Epi/mL) and TDC (5 × 10^5^ cells/mL) in the exponential growth phase were plated in a 96-well standard microplate in LIT medium (at 28 °C) and in D-MEM medium (at 37 °C in a 5% CO_2_ atmosphere), respectively. Both cultures were supplemented with 10% FBSi. For the trypanocidal assays, the EOs or their terpenes were added at varying concentrations (3.7 to 100 μg/mL) and incubated at the same culture conditions for 72 (Epi) or 24 h (Tryp). The growth inhibition was estimated by light microscopy through a differential count using the Trypan Blue (Gibco) dye exclusion technique. The results were expressed in terms of Inhibitory Concentration 50 (IC_50_) or the concentration at which parasite growth is inhibited by 50%. For Amas assays, a monolayer of Vero cells (3 × 10^5^ cel/mL) was infected with TDC in a 1:3 cell:parasite ratio and incubated for 24 h until Amas development occurred. Then, these intracellular forms were exposed for 120 h to EOs or terpenes in a treatment applied in two doses (at 0 and 48 h), under the same conditions described above. Growth inhibition analysis was assessed in Giemsa-stained films using light microscopy to determine the infected and uninfected cell percentage in a total of 300 cells. Cells without treatment and those treated with BNZ were used as negative and positive controls, respectively.

### Pharmacological interaction among terpenes on *T. cruzi*

A matrix of pharmacological interactions between limonene (the most selective terpene) and the other major EO terpenes was created for the three cyclic forms of *T. cruzi,* using the fixed-ratio isobologram method, as described previously by Fivelman et al. [34] with some modifications (Table 1). In the interaction matrix, the estimated IC_50_ for each terpene was used as fixed-value for the combinations. In addition, a mixture of limonene and BNZ was also evaluated.

**Table 1:** Interaction matrix among terpenes

Combination ID Number	Limonene (Compound X)	Compound Y
1	0.0	8× IC_50_
2	½ IC_50_	4× IC_50_
3	IC_50_	2× IC_50_
4	2× IC_50_	IC_50_
5	4× IC_50_	½ IC_50_
6	8× IC_50_	0.0

*IC*_*50*_ Inhibitory Concentration 50, *x* Number of times

The susceptibility evaluation was performed following the protocol described above for in vitro anti-parasitic activity. The Fractional Inhibitory Concentration (FIC) was calculated by: (Compound X (FIC) = Compound X (IC_50_) in combination) / (Compound X (IC_50_) alone); and the sum of FIC (ΣFIC) was determined by: ΣFIC = Compound X (FIC) + Compound Y (FIC). In this manner, synergistic, antagonist, or additive interactions were defined by ${}_{\overline{\mathrm{X}}}$ΣCIF < 1, ${}_{\overline{\mathrm{X}}}$ΣCIF > 1 or ${}_{\overline{\mathrm{X}}}$ΣCIF =1, respectively [31].

### Analysis of cell death

The death phenotype was analyzed by optical and fluorescent microscopy using phase contrast (fluorescence microscopy, Nikon Eclipse Ni). The cell morphology in Epi treated with two doses of the IC_50_ (2xIC_50_)*,* was examined by the 4 ‘,6diamidino-2, phenylindole probe (DAPI, 1 μg/mL, Sigma Aldrich) and a TUNEL assay (Molecular Probes, Invitrogen) for DNA fragmentation using a Terminal desoxynucleotidyl Transferase (TdT) label with d-UTP fluorescein. Determination of an oligonucleosomal-DNA ladder in treated parasites was also evaluated through DNA gel electrophoresis. Evaluation of the mitochondrial potential membrane in living parasites was performed with MitoTracker Red CMXRos (579 nm/599 nm emission/excitation wavelength) [35]. As a positive and negative apoptosis control, a 15 day-old Epi culture and an untreated fresh parasite culture were used, respectively.

### Flow cytometry analysis

For cell death characterization, an Annexin V/Dead with SYTOX® Green (Molecular Probes, Invitrogen) kit was used following the procedure specified in the manufacturer’s instructions. The phosphatidylserine externalization was determined by employing a recombinant Annexin V conjugated to the Orange Fluorescent phycobiliprotein R-PE, and to the necrotic cells using SYTOX™ Green nucleic acid stain. Briefly, 1 × 10^6^ Epi per mL were treated with 2xIC_50_ for 48 h, washed, and suspended in 1X Annexin-Binding Buffer. Next, R-PE Annexin V and SYTOX® Green Stain were added and incubated at 37 °C, 5% CO_2_, and 95% humidity, and analyzed in a FACSCanto II Flow Cytometer (provided by Dr. Marcos López from Fundación Cardiovascular de Colombia), with 488 nm/575 nm Excitation/Emission filters for R-PE and 503 nm/524 nm Excitation/Emission filters for SYTOX.

### Statistical analysis

Each treatment was tested in triplicate in three independent assays. The IC_50_ and CC_50_ were calculated by sigmoidal regression using the statistical software Msxlfit™ (ID Business Solution). The cytotoxicity analysis and statistically-significant difference determinations were performed using a Welch’s test for analysis of variances using SPSS 15.0 Software (IBM). Multiple comparison analysis was accomplished using a Tukey test with a 95% confidence level.

## Results

### Chemical composition and trypanocidal activity of *L. alba* EOs

**Fig. 1:**
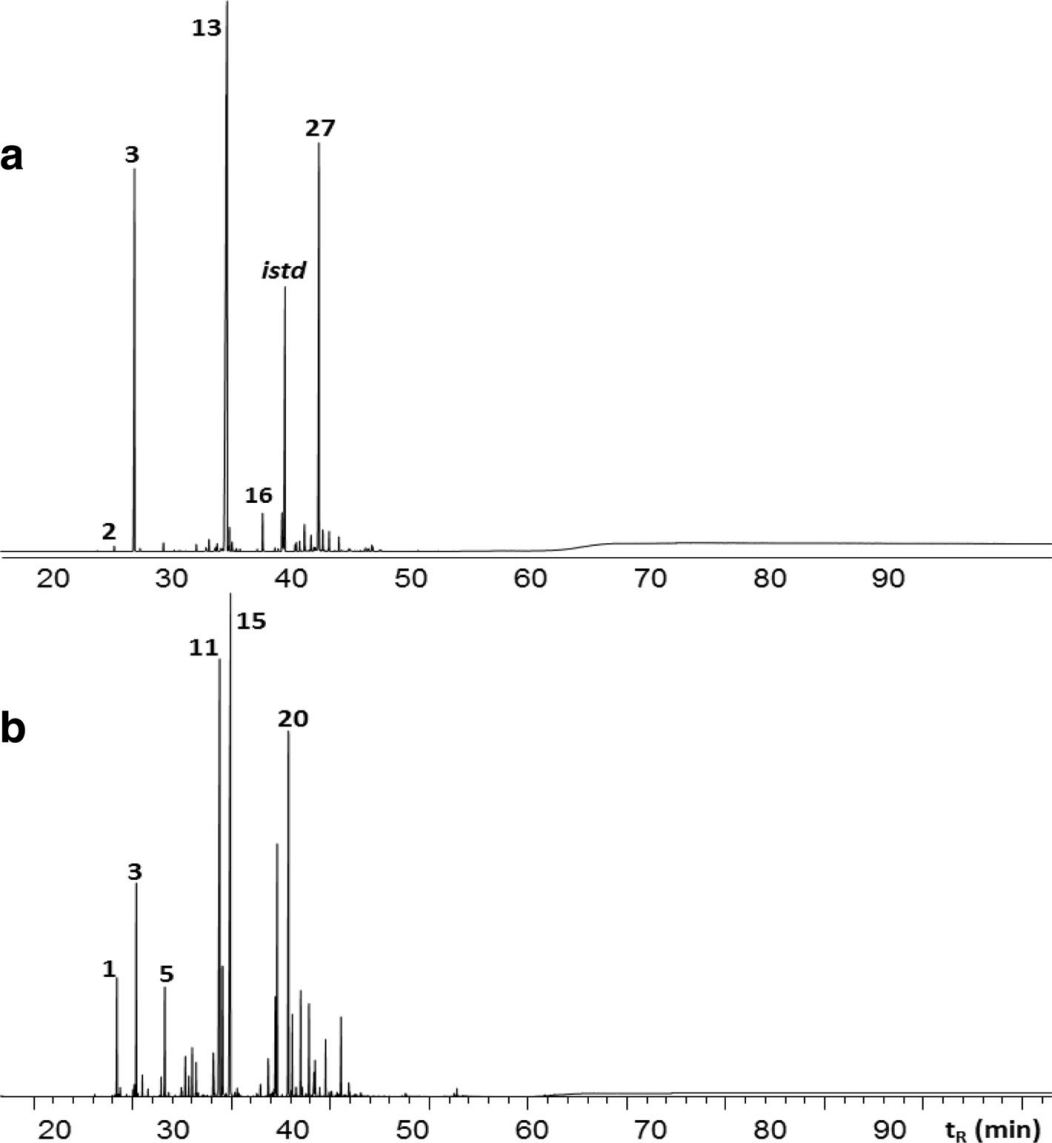
Typical gas chromatography-mass spectrometry (GC-MS) profiles, in a DB-5 (60 m) column with a mass selective detector (EI. 70 eV), of essential oils obtained from *Lippia alba* Carvone (**a**) and Citral (**b**) chemotypes by microwave-assisted hydrodistillation (MWHD). The corresponding peak identification is showed in Table 2

This work studied the trypanocidal properties of 12 EOs isolated from Citral and Carvone *L. alba* chemotypes produced under an array of standardized conditions for planting, collecting and extracting of the vegetal material. A typical chromatographic profile for each chemotype essential oil, obtained by mass spectra and linear retention indices, is showed in Fig. 1a and b, for Carvone and Citral oils, respectively. The corresponding peak assignment of these chromatograms are listed in Table 2.

**Table 2:** Peak assignment for GC-MS profiles of essential oils extracted by microwave-assisted hydrodistillation (MWHD) from *Lippia alba* Carvone (A) and Citral (B) chemotypes plants growing in Bucaramanga (Colombia)

Peak	Compound	LRI		Relative Quantity, %	
		DB-5MS^*a*^	DB-WAX^*b*^	Carvone (A)	Citral (B)
1	6-Methyl-5-hepten-2-one	986	1241	–	3.3
2	β-Myrcene	991	1064	0.8	–
3	Limonene	1034	1105	29.1	6.6
4	*trans*-β-Ocimene	1047	1153	0.7	0.2
5	Linalool	1100	1453	0.6	1.9
6	Citronellal	1154	1381	–	1.1
7	Borneol	1181	1613	0.8	–
8	*cis*-Dihydrocarvone	1203	1517	0.2	–
9	*trans*-Dihydrocarvone	1211	1537	0.2	–
10	Nerol	1231	1708	–	0.8
11	Neral	1248	1589	–	21.5
12	Geraniol	1252	1755	–	5.6
13	Carvone	1258	1653	35.0	–
14	Piperitone	1264	1641	2.4	–
15	Geranial	1275	1643	–	28.7
16	Piperitenone	1349	1842	4.0	–
17	Geranyl Acetate	1379	1662	–	1.5
18	β-Bourboneno	1396	1428	1.2	–
19	β-Elemene	1397	1496	1.0	3.0
20	*trans*-β-Caryophyllene	1436	1506	0.2	12.1
21	β-Gurjunene	1444	1447	0.2	–
22	α-Guaiene	1447	1498	–	1.8
23	*trans*-β-Farnesene	1456	1570	0.7	–
24	α-Humulene	1471	1580	0.1	2.7
25	γ-Gurjunene	1475	1587	0.4	–
26	Germacrene D	1486	1552	0.1	2.6
27	Bicyclosesquiphellandrene	1496	1624	8.2	–
28	Bicyclogermacrene	1509	1608	0.5	–
29	α-Bulnesene	1515	1627	–	1.4
30	Cubebol	1528	1855	0.5	–
31	Germacrene-4-ol	1591	1967	0.6	–
32	Caryophyllene Oxide	1600	1909	–	2.3

*LRI* Linear retention index

^a^Linear Retention Index experimentally determined in DB-5MS (60 m) column

^b^Linear Retention Index experimentally determined in DB-WAX (60 m) column

**Fig. 2:**
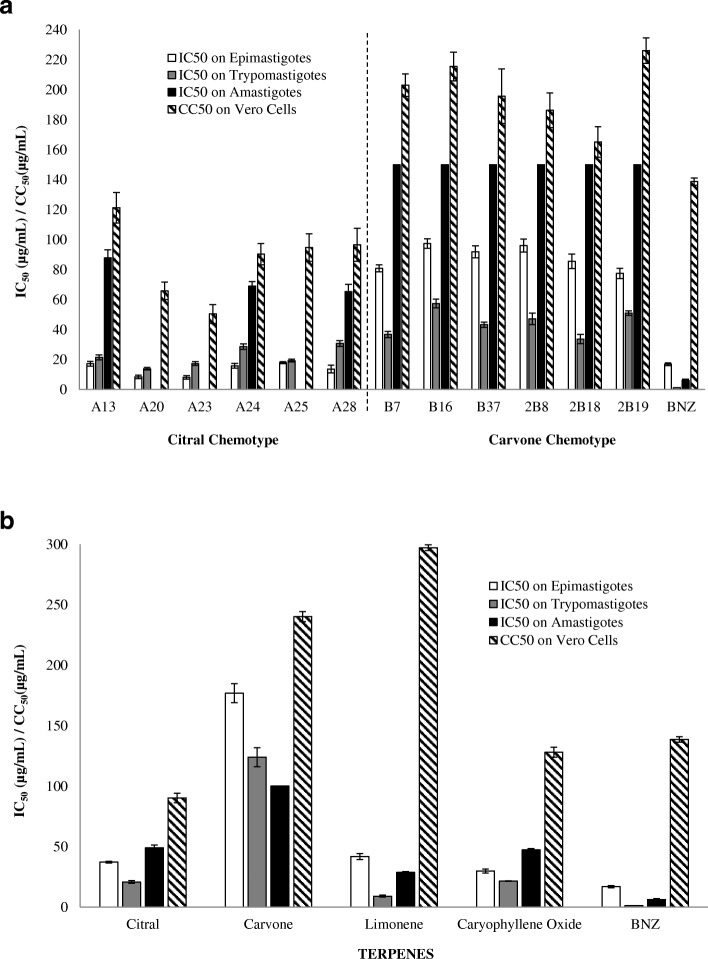
In vitro trypanocidal and cytotoxic activity of Citral and Carvone chemotype *L. alba* essential oils (**a**) and their bioactive terpenes (**b**) on cyclic forms of *Trypanosoma cruzi*. IC_50_: Inhibitory Concentration 50 on *T. cruzi*; CC_50_: Cytotoxic Concentration 50 on Vero Cells

All the EOs presented diversity in their chemical composition, and this variety also appeared as significant differences in their trypanocidal performance on the three cyclic forms of the parasite (Epi: F = 1320.080; *p* = 0.000; Tryp: F = 628.786; *p* = 0.000; Amas: F = 853.422; *p* = 0.000) (Tables 3 and 4, Fig. 2a).

**Table 3:** Relative chemical composition and anti-proliferative effect on *T. cruzi* of EOs extracted from the Citral chemotype of *L. alba*

Season	Material	EO^a^	Extra^b^ Time	Part Plant	Chemical Composition					Epi^f^		Tryp^j^		Amas^k^		Vero
			min^c^		Neral %	Geraniol %	Geranial %	Caryop^d^ %	CarOx^e^ %	IC_50_^g^ ± SD^h^ μg/mL	SI^i^	IC_50_^g^ SD^h^ μg/mL	SI^i^	IC_50_^g^ ± SD^h^ μg/mL	SI^i^	CC_50_^l^ ± SD^h^ μg/mL
Dry	Dry	A28	45	Infl^m^	19.3	31.5	31.3	2.3	–	14 ± 2.6	7.1	31 ± 1.9	3.1	66 ± 4.8	1.5	97 ± 11
Rainy	Fresh	A25	45	AL^n^	22.8	5.3	27.5	4.6	2.8	18 ± 0.7	5.3	19.4 ± 0.9	4.9	> 33.3	ND^r^	95 ± 9.2
	Dry	A13	30	YL^o^	30.6	–	54.5	2.9	–	17 ± 1.7	7.0	21 ± 1.6	5.7	88 ± 5.4	1.4	121 ± 10.1
		A20	30	ML^p^	32.1	–	54	4	2.4	9 ± 1.2	7.8	14 ± 0.9	4.7	> 33.3	ND	66 ± 5.9
		A23	90	ML	28	–	37.8	6.8	2.9	8 ± 1.3	6.2	17 ± 1.3	3.0	> 33.3	ND	51 ± 6.2
		A24	90	ML	24	–	34.3	2	5.7	16 ± 1.6	5.7	29 ± 1.8	3.1	69 ± 3.0	1.3	91 ± 7.1
		BNZ^q^	–	–	–	–	–	–	–	17 ± 0.9	8.2	1.2 ± 0.1	116.3	6 ± 0.9	22.4	139 ± 2.3

^a^*EO* Essential oil, ^b^*Extra* Extraction, ^c^*min* Minutes, ^d^*Caryop* Caryophyllene, ^e^*CarOx* Caryophyllene oxide, ^f^*Epi* Epimastigote, ^g^*IC*_*50*_ Inhibitory concentration 50, ^i^*SD* Standard deviation, ^i^*SI* Selectivity index (CC_50_/IC_50_), ^j^*Tryp* Trypomastigote, ^k^*Amas* Amastigote, ^l^*CC*_*50*_ Cytotoxic concentration 50, ^m^*Infl* Inflorescences, ^n^*AL* All leaves, ^o^*YL* Young leaves, ^p^*ML* Mature leaves, ^q^*BNZ* Benznidazole, ^r^*ND* Not determined

**Table 4:** Relative chemical composition and anti-proliferative effect on *T. cruzi* of EOs extracted from the Carvone chemotype of *L. alba*

Season	Material	EO^a^	Extra^b^ Time	Part Plant	Chemical Composition			Epi^f^		Tryp^j^		Amas^k^		Vero	
			min^c^		Limonene %	Carvone %	Piper^d^ %	BCE^e^ %	IC_50_^g^ ± SD^h^ μg/mL	SI^i^	IC_50_^g^ SD^h^ μg/mL	SI^i^	IC_50_^g^ ± SD^h^ μg/mL	SI^i^	CC_50_^l^ ± SD^h^ μg/mL
Dry	Fresh	B7	30	AL^m^	19.3	31.5	31.3	2.3	81 ± 2.4	2.5	37 ± 2.1	5.5	> 150	ND^o^	203 ± 7.5
Rainy	Dry	2B8	90	YL^n^	22.8	5.3	27.5	4.6	96 ± 4.4	1.9	47 ± 3.8	4.0	> 150	ND	186 ± 11.7
	Fresh	B16	90	AL	30.6	–	54.5	2.9	97 ± 3.2	2.2	57 ± 3.0	3.8	> 150	ND	216 ± 9.6
	Dry	B37	30	YL	32.1	–	54	4	92 ± 3.9	2.1	43 ± 1.8	4.5	> 150	ND	196 ± 18.2
	Dry	2B18	45	AL	28	–	37.8	6.8	86 ± 4.8	1.9	34 ± 3.1	4.9	> 150	ND	165 ± 10.2
	Dry	2B19	90	AL	24	–	34.3	2	78 ± 3.5	2.9	51 ± 1.4	4.4	> 150	ND	226 ± 8.5

^a^*EO* Essential oil, ^b^*Extra* Extraction, ^c^*min* Minutes, ^d^*Piper* Piperitenone, ^e^*BCE* Bicyclosesquiphellandrene, ^f^*Epi* Epimastigote, ^g^*IC*_*50*_ Inhibitory concentration 50; ^h^*SD* Standard deviation, ^i^*SI* Selectivity index (CC_50_/IC_50_), ^j^*Tryp* Trypomastigote, ^k^*Amas* Amastigote, ^l^*CC*_*50*_ Cytotoxic concentration 50, ^m^*AL* All leaves, ^n^*YL* Young leaves, ^o^*ND* Not determined

The best trypanocidal performance was observed in oils isolated from Citral chemotype plants, with IC_50_ values of 14 ± 1.5, 22 ± 1.4, and 74 ± 4.4 μg/mL on Epi, Tryp, and Amas, respectively (*p* < 0.05). Among these, the two lowest IC_50_ achieved were by A20 (9 ± 1.2 and 13.9 ± 0.9 μg/mL, on Epi and Tryp, respectively, *p* < 0.05) and A23 (8 ± 1.3 and 17 ± 1.3 μg/mL, on Epi and Tryp, respectively, *p* < 0.05) (Table 3, Fig. 2a). However, these oils also displayed a low selectivity, with high toxicity levels on Vero cells (A20: CC_50=_ 66 ± 5.9 μg/mL; A23: CC_50=_ 51 ± 6.2 μg/mL). Alternatively, oil A13 exhibited a significant level of anti-*T. cruzi* activity on the three cyclic forms (with IC_50_ of 17 ± 1.7 μg/mL, (SI = 7); IC_50_ 21 ± 1.6 μg/mL, (SI = 5.7); and IC_50_ 88 ± 5.4 μg/mL (SI = 1.4), on Epi, Tryp, and Amas, respectively); high cell death percentages (CDP) (Epi = 85 ± 1.7%; Tryp = 100%; Amas = 57 ± 3.1%; *p* < 0.05), at 100 μg/mL; and low toxic effect on mammal cells (CC_50_ 120 ± 10 μg/mL) (Table 3, Fig. 2a).

In contrast, EOs extracted from Carvone chemotype plants showed higher mean IC_50_ values (88 ± 3.7, 45 ± 2.5, and >  150 μg/mL on Epi, Tryp, and Amas, respectively (Table 4)), with a CDP under 60%, even at high concentrations (100 μg/mL), in both Epi (mean CDP of 56 ± 2.3%) and Tryp (mean CDP of 81 ± 3.1%) forms (Table 4). Among Carvone chemotype oils, the best trypanocidal activity was demonstrated by B7 with IC_50_ 81 ± 2.4 μg/mL, SI = 2.5, and a CDP of 60.1% on Epi forms; and IC_50_ 37 ± 2.1 μg/mL, SI = 5.5, and a CDP of 84.5%, on Tryp stages (*p* < 0.05) (Table 4, Fig. 2a). On *T. cruzi* replicative intracellular forms, none of the Carvone oils demonstrated significant activity (mean IC_50_ >  150 μg/mL; mean CDP 21 ± 4.4%). Nevertheless, on host cells (Vero) these EOs exhibited lower cytotoxicity (mean CC_50_ of 200 ± 11 μg/mL) than Citral oils (mean CC_50_ of 87 ± 8.3 μg/mL) (Table 4, Fig. 2a).

### Trypanocidal activity of *L. alba* Terpenes

For further studies using individual compounds, four of the major terpenes were selected from the *L. alba* EOs from both chemotypes, Citral (citral and (−) caryophyllene oxide) and Carvone (D (+) limonene and S (+) carvone). Table 5 presents the IC_50_ values obtained on the three studied parasitic forms and the CC_50_ values estimated on Vero cells.

**Table 5:** Anti-parasitic effect on *Trypanosoma cruzi* of the major terpenes of Citral and Carvone chemotype *L. alba* essential oils

Terpenes	Epi^a^ IC_50_^b^ ± SD^c^ (μg/mL)	SI^d^	Tryp^e^ IC_50_^b^ ± SD^c^ (μg/mL)	SI^d^	Amas^h^ IC_50_^b^ ± SD^c^ (μg/mL)	SI^d^	Vero CC_50_^i^ ± SD^c^ (μg/mL)
CarOx^j^	30 ± 1.7	4.3	22 ± 0.3	5.9	47 ± 1.0	2.7	128 ± 4.2
Limonene	42 ± 2.5	7.1	9 ± 0.8	32.8	29 ± 0.7	10.3	297 ± 2.4
Citral	37 ± 0.7	2.4	21 ± 1	4.3	49 ± 2.3	1.8	90 ± 3.9
Carvone	177 ± 7.9	1.4	124 ± 8	1.9	> 100	ND^l^	240 ± 4.1
BNZ^k^	17 ± 0.9	8.2	1.2 ± 0.1	116.3	6.2 ± 0.9	22.4	139 ± 2.3

^a^*Epi* Epimastigote, ^b^*IC*_*50*_ Inhibitory concentration 50, ^c^*SD* Standard deviation, ^d^*SI* Selectivity index (CC_50_/IC_50_), ^g^*Tryp* Trypomastigote, ^h^*Amas* Amastigote, ^i^*CC*_*50*_ Cytotoxic concentration 50, ^j^*CarOx* Caryophyllene oxide, ^k^*BNZ* Benznidazole, ^l^*ND* Not determinated

Among the studied terpenes, D (+) limonene exhibited the best IC_50_ on Tryp (IC_50_ 9 ± 0.8 μg/mL, SI = 32.8, *p* < 0.05), and Amas (IC_50_ 29 ± 0.7 μg/mL, SI = 10.3, *p* < 0.05) forms, (Table 5, Fig. 2b), with the most selective and the least toxic performance on mammal cells (CC_50_ 297 ± 2.4 μg/mL, and SI = 7.1, *p* < 0.05), with a CC_50_ even lower than the reference drug (BNZ: CC_50_ 139 ± 2.3 μg/mL). At the other end of the spectrum, S (+) carvone constituted the terpene with the worst trypanocidal activity on all evolutionary *T. cruzi* forms (Epi: IC_50_ 177 ± 7.9 μg/mL, and SI = 1.4; Tryp: IC_50_ 124 ± 7.8 μg/mL, and SI = 1.9; Amas: IC_50_ >  100 μg/mL) (Table 5). It is worth pointing out that all the terpenoid fractions, except carvone, were able to induce significant cell death on extracellular forms of the parasite at 50 μg/mL (Epi: CDP = 66 ± 1.9%; Tryp: CDP = 90 ± 1.2%, *p* < 0.05) with caryophyllene oxide being the terpene with the highest rate of death on Epi (78 ± 2.3%), and Tryp (98 ± 0.5%, *p* < 0.05).

### Terpene pharmacological interactions on *T. cruzi*

**Fig. 3:**
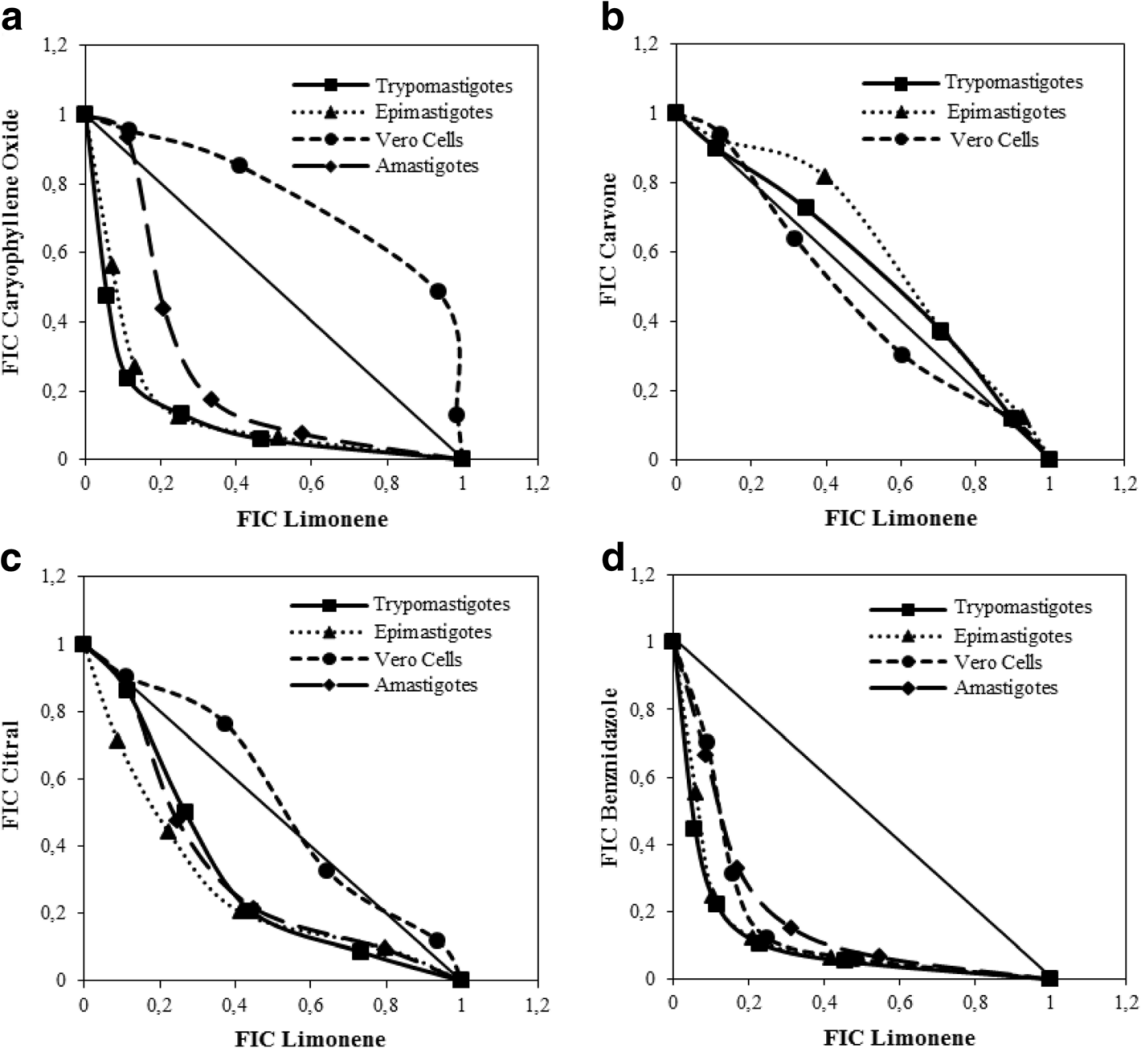
Pharmacological interaction isobolograms among major terpenes of the Citral and Carvone chemotype *L. alba* essential oils: **a** limonene with caryophyllene oxide; **b** limonene with carvone; **c** limonene with citral; and **d** limonene with Benznidazole (BNZ). The interaction tests were performed on Epimastigotes, Trypomastigotes and Amastigotes of *T. cruzi* and Vero cells. Dotted lines correspond to an additive effect; points below, on, and above line indicate a synergistic, additive, and antagonistic effect, respectively

Because D (+) limonene demonstrated the best performance as a selective trypanocidal agent on all the evolutionary forms of *T. cruzi*, this monoterpene was selected as a fixed-compound of a pharmacological interaction matrix among terpenes and BNZ. This matrix was assembled taking the IC_50_ values determined previously (Table 5). Following the FIC value interpretation described by Azeredo and Soares, (2013) [31], all the evaluated interactions were found to be synergic on both extra and intracellular forms of the parasite (except limonene with carvone, with ΣFIC = 1.10 and 1.04, on Epi and Tryp, respectively) (Table 6). Figure 3 shows these pharmacological relations as isobolograms of the mean FIC of each combination. The highest synergy was exhibited by limonene/BNZ combinations (Epi: ΣFIC = 0.44; Tryp: ΣFIC = 0.42; Amas: ΣFIC = 0.58) (Table 6, Fig. 3), with the best trypanocidal performance achieved by the 4xIC_50_ limonene:½IC_50_ BNZ mixture (4 times IC_50_ limonene plus one half of the IC_50_ of BNZ), which reduced by 14, 16, and 17 times the BNZ IC_50_ on Amas, Epi, and Tryp, respectively. Despite its good performance, this combination also resulted in an increased cytotoxicity on Vero cells (ΣFIC = 0.54). On the other hand, limonene with caryophyllene oxide represented the second-best combination by anti-parasitic efficacy (Epi Σ FIC = 0.49; Tryp Σ FIC = 0.45; and Amas Σ FIC = 0.71), while offering an additional advantage of reduction of the individual cytotoxicity of each terpene on Vero cells (ΣFIC = 1.22) (Table 6, Fig. 3).

**Table 6:** Pharmacological interactions among terpenes derived from *L. alba*

Parasitic Form	Limonene + Compound	ΣFIC^f^ μg/mL ± SD^g^	Pharmacological interaction
Epi^a^	CarOx^d^	0.5 ± 0.13	Synergism
	Carvone	1.1 ± 0.08	Antagonism
	Citral	0.7 ± 0.13	Synergism
	BNZ^e^	0.4 ± 0.13	Synergism
Tryp^b^	CarOx^d^	0.4 ± 0.10	Synergism
	Carvone	1.04 ± 0.04	Antagonism
	Citral	0.6 ± 0.10	Synergism
	BNZ^e^	0.4 ± 0.10	Synergism
Amas^c^	CarOx^d^	0.7 ± 0.23	Synergism
	Carvone	ND^h^	ND^h^
	Citral	0.8 ± 0.15	Synergism
	BNZ^e^	0.6 ± 0.13	Synergism
Vero	CarOx^d^	1.2 ± 0.16	Antagonism
	Carvone	1.0 ± 0.07	Additive
	Citral	1.0 ± 0.07	Antagonism
	BNZ^e^	0.5 ± 0.18	Synergism

^a^*Epi* Epimastigote, ^b^*Tryp* Trypomastigote, ^c^*Amas* Amastigote, ^d^*CarOx* Caryophyllene oxide, ^e^*BNZ* Benznidazole, ^f^*FIC* Fractional inhibitory concentration, ^g^*SD* Standard deviation, ^h^*ND* Not determinated

### Morphological analysis on *T. cruzi* forms

**Fig. 4:**
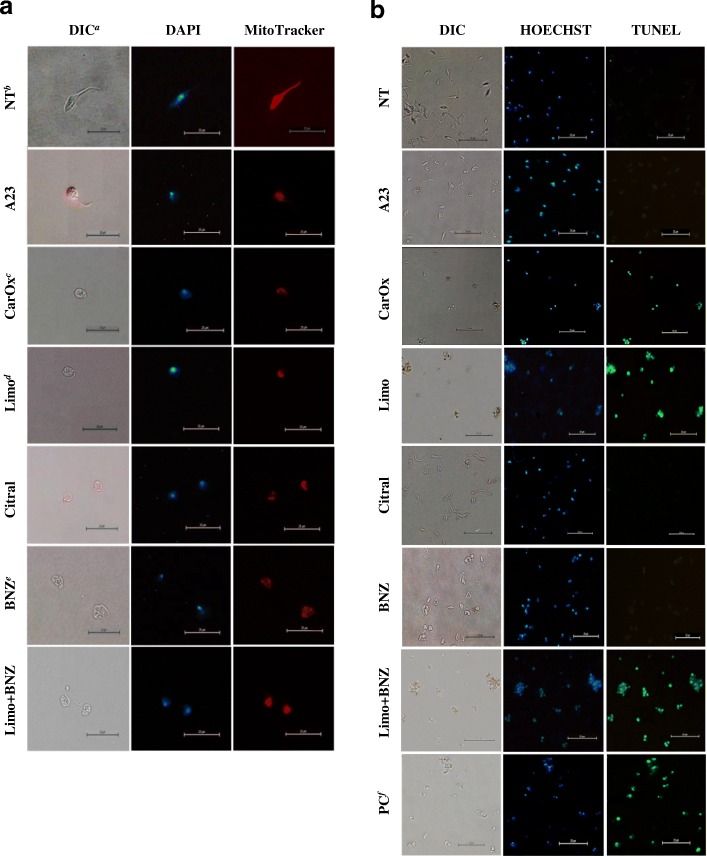
Cell morphology changes of *Trypanosoma cruzi* by fluorescent and optical microscopy. **a** Cell morphology, mitochondrial membrane potential, nuclear and kinetoplast DNA of *T. cruzi* epimastigotes after treatment with essential oils, terpenes, or BNZ. **b** DNA fragmentation analysis by TUNEL assay on *T.cruzi* epimastigotes treated with terpenes. The preserved parasitic DNA was visualized with a blue HOECHST fluorescent probe (negative TUNEL) and the free DNA strands were observed in green (positive TUNEL). ^a^DIC: Differential Interference Contrast Microscopy; ^b^NT: No Treatment; ^c^CarOx: caryophyllene oxide; ^d^Limo: limonene; ^e^BNZ: Benznidazole; ^f^PC: Positive control: DAPI: cells stained with DAPI nuclear fluorescent stain observed in UV filter. MitoTracker: cells stained with MitoTracker Red CMXRos stain observed in an Excitation/Emission 579/599 (nm) filter. Photographs are representative of 10 observed fields

The morphological changes induced by the treatments studied (EOs, terpenes, or BNZ, and their combinations) were analyzed by optical and fluorescent microscopy using phase contrast, and nuclear specific (DAPI) and mitochondrial membrane potential (Mitotracker Red CMXRos [35]) stains. As shown in Fig. 4, some of the tested treatments induced significant changes on parasitic morphology such as: spherical cell conformation, reduced cytoplasmic volume (Fig. 4a, DIC), mitochondrial membrane potential deplection (Fig. 4a, MitoTracker), and formation of a nuclear speckled/condensation pattern (Fig. 4a, DAPI). In one unique finding, the caryophyllene oxide treatment also caused a flagellum to be lost. Conversely, *T. cruzi* cells treated with BNZ displayed cellular edema and loss of cellular membrane integrity, but with conserved mitochondrial energetic potential (Fig. 4a). Under the same conditions, Vero host cells did not present visible morphological alterations (data not shown).

### DNA fragmentation

A possible endonuclease activation triggered by studied compounds (oils, terpenes or their combinations) was assessed through agarose gel DNA electrophoresis and TUNEL analyses. DNA degradation was observed by band disappearance in agarose gel (data not shown) and confirmed through green fluorescence on nuclei and kinetoplasts from Epi forms treated for 48 h with double doses at IC_50_ (2xIC_50_) of limonene, caryophyllene oxide, and the mix limonene:BNZ; with percentages of 94, 99, and 98, respectively (Fig. 4b). Non-significant fragmentation was observed on untreated Epi (Fig. 4b).

### Phosphatidylserine externalization

**Fig. 5:**
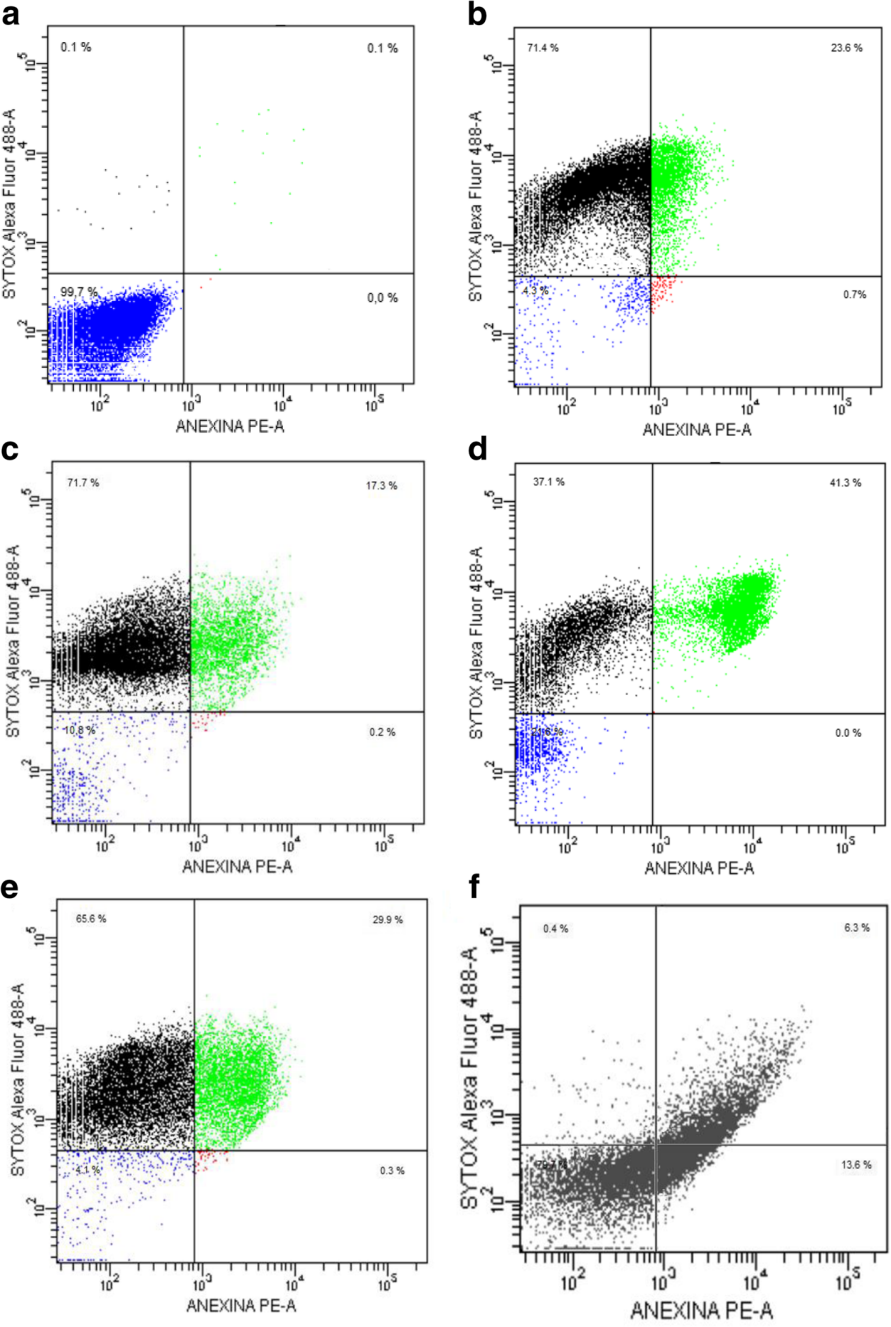
Flow cytometry analysis of phosphatidylserine externalization of *T. cruzi* epimastigotes treated with terpenes or Benznidazole. **a** Negative control (untreated culture). **b** caryophyllene oxide; **c** limonene; **d** citral; **e** limonene:BNZ; and **f** apoptosis positive control (15 day-old parasite culture). The flow cytometry histograms are representative of two independent experiments

A flow cytometry analysis was carried out to determine the general mechanism of cell death. As expected, untreated Epi showed high viability rates (99.7%) (Fig. 5a), whereas the various terpene treatments (48 h at 2xIC_50_) caused high percentages of cell death, with 95.7, 89.2, 78.4, and 95.9% for cayophyllene oxide, limonene, citral, and the combination limonene:BNZ, respectively (*p* = 0.0001). No treatment showed statistically significant levels of negative SYTOX + positive Annexin V (*p* > 0.05), except the apoptosis positive control (15 day-old parasite culture) (13.6%) (Fig. 5f). On the other hand, all the treated cultures displayed high percentages of positive SYTOX and positive Annexin V. These results suggest a possible trigger of a late apoptosis mechanism (Fig. 5).

## Discussion

In Chagas Disease, the pathogen-specific treatments – such as BNZ – should be prescribed for acute cases and for younger patients with little or no evidence of established cardiomyopathy [36, 37]. On the other hand, recent results from global trials have questioned the benefit of these therapies in chronic patients [8, 36]. In the case of BNZ, the drug demonstrated a significant decrease of the circulating parasite load, but no substantial effect in the prevention of the clinical decline [8, 36]. Therefore, most patients with advanced *T. cruzi* disease receive only symptomatic treatment for cardiomyopathy or digestive symptoms. This absence of an association between parasite clearance by BNZ and the clinical progression of heart disease has been ascribed to both the restricted activity of the treatment in the inflammatory and fibrotic cardiomyopathy lesions, as well as the irreversibility of this damage [36]. Thus, alternative approaches for Chagas infection management should aim to control not only the parasite load, but also all the factors associated with cardiomyopathy progression (oxidative stress and immune effectors, among others) [36, 38].

Since parasitic protozoa are very sensitive to oxidative stress [39], the most common trypanocidal and anti-chagasic drugs like Nitroimidazoles derived (BNZ) and Nitrofurans (NFX) were developed based on their capability to induce Reactive Oxygen Species (ROS) production [40]. However, the clinical use of both medicines has been limited due to their high toxicity [41], mutagenic potential [42], the severity of their side effects [38, 41], and the lack of significant effects on clinical disease progression in the late stages of *T. cruzi* infection [8, 37].

In general, the development of new trypanocidal agents has been focused on the use of molecules that alter the cellular redox potential and take advantage of the scarce antioxidant defenses of the parasite [39, 40]. A similar anti-protozoal effect has been described for essential oils rich in terpenes extracted from aromatic plants [43]. In this regard, EOs derived from *Cymbopogon citratus* showed promising results, with low IC_50_ values against *T. cruzi* (15.5 μg/mL for Epi and Tryp; and 5.1 μg/mL for Amas) [17]. These trypanocidal effects were attributed to the high levels of the oxygenated monoterpene citral (a mixture of neral and geranial) [17]. Similar outcomes were obtained with oils extracted from a Colombian (Santander) variant of *L. alba* (Citral chemotype), which were rich in such terpenes as citral, geraniol, timol, and caryophyllene oxide [30].

In this work, we tested the trypanocidal and cytotoxic activity of 12 EOs, derived from two different plant chemotypes (Citral and Carvone) of *L. alba,* which were produced under controlled conditions of growth (geographic location, cultivation environment, and soil), plant parameters (age and part), material state (fresh or dry) and extraction conditions (time). These standardized procedures were prepared taking into consideration the recognized high phenotypical plasticity of the plant in response to genetic, environmental, and production parameters [25], which can induce significant variations in its EO constituents, as well as in their biological activities [25, 44].

In this regard, our results also confirmed significant differences in the major chemical compounds (terpenes), and trypanocidal activity of the *L. alba* oils produced under varying parameters (Tables 3 and 4, and Fig. 2a). While mixtures rich in citral and caryophyllene oxide (Citral chemotype EOs) achieved good performance on extracellular forms of *T. cruzi* (mean IC_50_ values of 13.6 and 21.9 μg/mL on Epi and Tryp, respectively) (Table 3); EOs of the same plant but rich in carvone and limonene (Carvone chemotype), displayed poor inhibitory results (mean IC_50_ values of 88.2 and 44.9 μg/mL for Epi and Tryp, respectively) (Table 4). Individual assays with a solution of citral consistently found that this compound caused an efficient arrest of the parasite’s growth with IC_50_ values of 37.2 and 20.8 μg/mL on Epi and Tryp, respectively (Table 5).

With respect to citral, several studies on cancer and immune cell line models have demonstrated its interesting biological properties. As an anti-tumoral, this monoterpene exhibited significant anti-proliferative effects, in vitro and in vivo [45–48] and this cell growth inhibition was ascribed to the induction of mitochondrial apoptosis pathways (through p53 activation triggered by an ROS increase) [45]. In addition, citral has also been described as anti-inflammatory agent. In lipopolysaccharide (LPS)-stimulated macrophages, this terpene was found to suppress the expression of pro-inflammatory markers such as NLRP2 (NLR Family Pyrin Domain Containing 2), Interleukin (IL) 6 and IL-1β [24], Tumoral Necrosis Factor (TNF) α [49], as well as to activate the Peroxisome Proliferator-Activated Receptor (PPAR) γ dependent-Cyclooxygenase 2 (COX2) promotor [50]. It is worth mentioning that over a range of different cells, the expression of COX2 is regulated in a variety of ways; playing an important role in tumoral genesis, inflammation, development, and circulatory homeostasis. In these activated macrophages, citral also blocks the genic expression of the LPS-induced Nitric Oxide Synthase (iNOS) [51] and, consequently, the production of Nitric Oxide (NO). It is thought that this inhibition could suppress transcriptional activation and the translocation of the nuclear factor-kappa B (NF-ƙB). These results suggest that citral is an anti-inflammatory agent whose effects could be associated with NF-ƙB suppression [51], indicating that this compound may be a promising candidate for the treatment of inflammatory conditions like Chagas Disease.

Similarly, anticancer and anti-inflammatory properties have also been attributed to caryophyllene oxide, another major and bioactive constituent of Citral chemotype *L. alba* EOs. On human prostate and breast cancer cells, this sesquiterpene isolated from the EOs of medicinal plants such as guava (*Psidium guajava*), oregano (*Origanum vulgare* L.), cinammon (*Cinnamomum spp*.), clove (*Eugenia caryophyllata*), and black pepper (*Piper nigrum L*.), inhibited constitutive survival pathways (PI3K/AKT/mTOR/S6 K1) and ROS-dependent MAPK activation during tumorigenesis; triggering apoptosis on tumoral lineages and preventing inflammation, angiogenesis, and metastasis [52]. Furthermore, on stimulated primary splenocytes, caryophyllene oxide significantly increases the Th2/Th1 coefficient [22].

In our work, caryophyllene oxide was correlated with a trypanocidal effect, being found to be one of the major components of the most trypanocidal EOs studied: A23 and A20 (*L. alba* citral chemotype). Similarly, Cheikh Ali et al.*,* [18], found a minimal lethal concentration of 0.1 μg/mL for EOs extracted from *Aframomum sceptrum* on cyclic forms of *T. brucei*; this trypanocidal action being associated with the presence of caryophyllene oxide. In the present study, this compound demonstrated a significant anti-proliferative effect against *T. cruzi* Epi (IC_50_ = 29.8 μg/mL), Tryp (IC_50_ = 21.6 μg/mL), and Amas (IC_50_ = 47.4 μg/mL) (Table 5).

Another major *L. alba* terpene studied herein was limonene. This monoterpene is one of the main components of the Carvone chemotype oils. Due its beneficial pharmacological characteristics such as: low toxicity (used as food additive for decades) [53], high bioavailability [54], and selective anti-tumoral effect on a variety of cancer cell lines (leukemia, lymphoma, prostate, hepatic, colorectal, pancreatic, gastric, and breast, among others [54–57]); several research efforts have been undertaken with respect to this monoterpene. Interestingly, on a prostate cancer model, limonene caused apoptotic programmed cell death by the induction of a selective oxidative stress on tumoral cells [57]. As for protozoa, cancer cells are highly vulnerable to cell death induced by pro-oxidant agents (such as limonene) due their high metabolism and their deficient antioxidant mechanisms [58]. In this study, limonene was the best trypanocidal and most selective terpene with the lowest inhibitory doses (IC_50_ of 9.0, 28.7, and 41.8 μg/mL on Tryp, Amas, and Epi, respectively). However, this good performance was not replicated when *T. cruzi* forms were treated with oils rich in carvone and limonene (*L. alba* Carvone chemotype EOs displayed higher IC_50_ levels on Epi = 88.2 μg/mL, Tryp = 44.9 μg/mL, and Amas > 150 μg/mL) (Table 5). Interestingly, carvone was the least effective trypanocidal terpene, with the lowest values of cell death percentage induction and highest IC_50_ results (Table 5). These results were associated with the strong antioxidant capability previously ascribed to this monoterpene [59]. Accordingly, a possible antagonism seems likely between limonene and carvone (which was present in the oil mixture in levels close to 42%) (Table 4). Pharmacological interaction tests confirmed that the presence of carvone in the *L. alba* Carvone chemotype oils, impaired the limonene’s trypanocidal performance on *T. cruzi* Tryp (ΣCIF: 1.04 μg/mL) and Epi (ΣCIF: 1.10 μg/mL) forms (Table 6). In further assays, limonene presented a synergistic pharmacological interaction with the other bioactive *L. alba* terpenes (citral and caryophyllene oxide), and with BNZ, exhibiting ΣFIC values < 0.8 μg/mL on the three parasite forms analyzed (Table 6). In combination with BNZ, limonene caused a significant decrease of the BNZ-IC_50_, by 14, 16, and 17 times on Amas, Epi, and Tryp, respectively (Table 6).

These positive interactions could have been due to the simultaneal action of these compounds on diverse and additive mechanisms that lead to cell death in susceptible lineages. Such mechanisms may include: a) polymerization microtubules disruption (citral) [60]; b) endoplasmic reticulum stress induction (citral) [60]; c) PIP3/AKT survival pathway inhibition (limonene, citral, and caryophyllene oxide) [52, 61, 62]; d) oxidative stress stimulation (limonene, citral, and caryophyllene oxide) [52, 57, 63]; and e) apoptosis by caspases activation (citral and limonene) [61–63], among others.

It is important to add that the parasite cells treated for 48 h with some of the studied compounds (A23 oil, citral, caryophyllene oxide, limonene, and the mixture of limonene and BNZ) evidenced typical characteristics of apoptosis, such as cytoplasmic blebbing, cell shrinkage, flagellum absence, loss of mitochondrial membrane potential, condensation of the nuclear chromatin, and DNA fragmentation (Fig. 4a). Also, the treatment of parasites with caryophyllene oxide or limonene (alone, in combination, or with BNZ) led to positive results in TUNEL assays (Fig. 4b). Correspondingly, an impairment of membrane potential (Fig. 4) and the externalization of phosphatidylserine (Fig. 5) were observed on *T. cruzi* cells treated with citral, limonene, and caryophyllene oxide. These results suggest a possible activation of an early apoptosis mechanism that rapidly progresses to late apoptosis (positive SYTOX plus positive Annexin V) accompanied by DNA fragmentation. In trypanosomatids like *Leishmania donovani, T. brucei*, and *T. cruzi,* these same characteristics have been reported in parasites suffering calcium imbalance and oxidative stress (by ROS) [64], mitochondrial enzyme knockdown [65], or treatment with sterols [66]. In these studies, the previously mentioned features were associated with a possible programmed cell death such as apoptosis or autophagy [67].

In an illness with a complex pathogenesis like Chagas Disease (which involves the parasitic persistence that triggers and sustains an anti-inflammatory immune response), the use of synergic drugs (like limonene/caryophyllene oxide) with several biological advantages (significant trypanocidal activity [32], low toxicity on mammal tissues [53]; and anti-genotoxic [68], chemoprotective [23], and anti-inflammatory activity [22]) could be an interesting platform for the development of an adjuvant therapy that enhances the therapeutic effects of the conventional treatments, principally in advanced stage of the infection (probably improving trypanocidal action, reducing therapeutic doses, increasing tolerance, or retarding resistance development) [69].

## Conclusions

In this work, a range of growth, plant, and extraction parameters were found to significantly influence the chemical composition and trypanocidal activity of essential oils isolated from *L. alba* Citral and Carvone chemotypes. *L. alba* Citral chemotype oils, extracted under known and controlled conditions, presented significant trypanocidal activity on three cyclic *T. cruzi* forms: epimastigotes, trypomastigotes, and amastigotes. Assays using pure solutions of the main terpenes that constitute *L. alba* EOs, confirmed an association among parasitological activity and the presence of citral and caryophyllene oxide. Tests using EOs extracted from Carvone chemotype (rich in carvone and limonene), and their most important components, established an antagonistic relationship between carvone and limonene in their trypanocidal performance. Nevertheless, in this study, the best anti-*T. cruzi*, and most selective, activity was achieved by limonene. Citral, caryophyllene oxide, and limonene exhibited the induction of a possible apoptotic-like phenotype. In the synergistic interaction tests, limonene also improved the trypanocidal performance of citral, caryophyllene oxide, and even BNZ, on the three parasitic forms studied. The best synergic terpene activity was displayed by the limonene and caryophyllene oxide combination. These results should be confirmed by further pre-clinical studies and could be of interest for the development of alternative and adjuvant treatments improving the tolerance and parasitological efficacy, and broadening the spectrum of the effects, of the current conventional therapies for late phases of Chagas Disease. In such research, *L. alba* EOs represent a renewable source for commercial exploitation of these terpenes.

## Abbreviations

ΣFIC: Sum FIC
AL: All Leaves
Amas: Amastigote
ANOVA: Analysis of variance
BCE: Bicyclosesquiphellandrene
BNZ: Benznidazole
CarOx: Caryophyllene oxide
Caryop: Caryophyllene
CC_50_: Cytotoxic Concentration 50
CDP: Cell death percentages
CENIVAM: National Research Center for Agroindustrialization of Aromatic Medical and Tropical Species
COX2: Cyclooxygenase 2
DAPI: 4′,6-diamidino-2-phenylindole
DMEM: Dulbecco’s Modified Eagle’s Medium
DMSO: Dimethyl sulfoxide
DNA: Deoxyribonucleic acid
EO: Essential oil
Epi: Epimastigotes
FBS_i_: Inactivated Fetal Bovine Serum
FIC: Fractional Inhibitory Concentration
GC-MS: Gas chromatography coupled mass spectrometry
IC_50_: Inhibitory Concentration 50
IL: Interleukin
Inf: Inflorescences
iNOS: Induced Nitric Oxide Synthase
*L. alba*: *Lippia alba*
LIT: Liver Infusion Tryptose
LPS: Lipopolysaccharide
Min: Minutes
ML: Mature leaves
mm: Millimeter
MWHD: Microwave-Assisted Hydrodistillation
ND: Not determined
NF-ƙB: Nuclear factor-kappa B
NFX: Nifurtimox
NLRP2: NLR Family Pyrin Domain Containing 2
nm: Nanometer
NO: Nitric Oxide
NTD: Neglected Tropical Disease
OD: Optical Density
PBS: Phosphate Buffered Saline
Piper: Piperitenone
PPAR: Peroxisome Proliferator-Activated Receptor
ROS: Reactive species of oxygen
RPMI: Roswell Park Memorial Institute
SD: Standard deviation
SI: Selectivity index
*T. brucei*: *Trypanosoma brucei*
*T. cruzi*: *Trypanosoma cruzi*
TDC: Trypomastigotes Derived from Cells
TdT: Terminal desoxynucleotidyl Transferase
TNF: Tumor Necrosis Factor
Tryp: Trypomastigote
UTP: Uridine Triphosphate
YL: Young leaves
